# Surfactin and fengycin contribute to the protection of a *Bacillus subtilis* strain against grape downy mildew by both direct effect and defence stimulation

**DOI:** 10.1111/mpp.12809

**Published:** 2019-05-18

**Authors:** Yan Li, Marie‐Claire Héloir, Xun Zhang, Mareen Geissler, Sophie Trouvelot, Lucile Jacquens, Marius Henkel, Xin Su, Xuewen Fang, Qi Wang, Marielle Adrian

**Affiliations:** ^1^ Department of Plant Pathology, College of Plant Protection China Agricultural University Beijing 100193 P. R. China; ^2^ Agroécologie, AgroSup Dijon, CNRS, INRA, Univ. Bourgogne, Univ. Bourgogne Franche‐Comté Dijon F‐21000 France; ^3^ Institute of Food Science and Biotechnology, Department of Bioprocess Engineering University of Hohenheim Fruwirthstrasse 12 Stuttgart 70599 Germany

**Keywords:** *Bacillus subtilis*, defence, downy mildew, fengycin, lipopeptide, *Plasmopara viticola*, surfactin

## Abstract

*Bacillus subtilis* GLB191 (hereafter GLB191) is an efficient biological control agent against the biotrophic oomycete* Plasmopara viticola*, the causal agent of grapevine downy mildew. In this study, we show that GLB191 supernatant is also highly active against downy mildew and that the activity results from both direct effect against the pathogen and stimulation of the plant defences (induction of defence gene expression and callose production). High‐performance thin‐layer chromatography analysis revealed the presence of the cyclic lipopeptides fengycin and surfactin in the supernatant. Mutants affected in the production of fengycin and/or surfactin were thus obtained and allowed us to show that both surfactin and fengycin contribute to the double activity of GLB191 supernatant against downy mildew. Altogether, this study suggests that GLB191 supernatant could be used as a new biocontrol product against grapevine downy mildew.

## Introduction

Grapevine (*Vitis vinifera* cvs), a major fruit crop worldwide, is susceptible to many cryptogamic diseases such as downy mildew (*Plasmopara viticola*), powdery mildew (*Erysiphe necator*) and grey mould (*Botrytis cinerea*). All of these can cause severe economic losses in both wine and table grape production. To ensure the quantity and quality of the harvest, the use of chemical fungicides remains the most common protection strategy. However, there is an increasing societal demand for human healthiness and environmentally friendly crop disease management. Alternative/complementary strategies to chemicals, such as biological control, have therefore been developed (Compant and Mathieu, 2016).

Biocontrol products protect plants against pathogens via diverse mechanisms such as direct antagonistic effect and/or activation of plant defences (Landy *et al.*, 1948; Perazzolli *et al.*, 2011; Shafi *et al.*, 2017). They have various origins, including more or less purified plant extracts (Krzyzaniak *et al.*, 2018) as well as living non‐pathogenic microorganisms (i.e. biological control agents, BCA) and their metabolites (Abdalla *et al.*, 2011; Harwood *et al.*, 2018; Ongena and Jacques, 2008). Studies have shown members of the *Bacillus* genus can be used as a versatile weapon against plant pathogens (Jacobsen *et al.*, 2004; McSpadden Gardener and Fravel, 2002; Pérez‐García *et al.*, 2011; Shafi *et al.*, 2017). *Bacillus* is ubiquitous and widely distributed in both water and terrestrial ecosystems and even in environments under extreme conditions due to its ability to form endospores, which provide it with resistance and enhance its viability in different environmental conditions (Harwood *et al.*, 2018; Nicholson, 2002; Villarreal‐Delgado *et al.*, 2018). This is a technological advantage for the dryness step required for formulation into stable products and long shelf‐life (Keswani *et al.*, 2016; Schisler *et al.*, 2004). As a biocontrol agent, *Bacillus* uses various modes of action against different plant pathogens such as antagonism, competition for niche space and nutrients, and induction of host resistance (Santoyo *et al.*, 2012; Villarreal‐Delgado *et al.*, 2018). *Bacillus*‐based BCAs therefore represent a large range of microbe products used for crop protection (Fravel, 2005; Shafi *et al.*, 2017).

*B. subtilis* is one of the most commercialized BCAs. It produces various bioactive compounds against a broad spectrum of pathogens. Some of the most prominent bioactive compounds for plant protection are cyclic lipopeptides (CLPs) (Stein, 2005). *B. subtilis* CLPs include the surfactin, iturin and fengycin (or plipastatin) families and they have different activities (Falardeau *et al.*, 2013; Stein, 2005). Members of the surfactin family are biosurfactant molecules with antiviral and antibacterial activities but no marked fungitoxicity (Falardeau *et al.*, 2013). For example, surfactin plays a major role in suppressing bacterial fruit blotch, but does not seem to have a direct toxic effect on *Botrytis* (Fan *et al.*, 2017b; Farace *et al.*, 2015). Besides the direct antagonistic activity, the surfactin family also induces resistance on a diversity of hosts against various diseases by stimulating plant immune responses. For example, purified surfactin triggers defence responses in grapevine and tobacco cell suspensions (Farace *et al.*, 2015; Jourdan *et al.*, 2009) and induces systemic resistance (ISR) against *B. cinerea* in tomato plants (Cawoy *et al.*, 2014). The fengycin family mostly shows antifungal activity (Deleu *et al.*, 2008). Unlike surfactin, fengycin‐induced defences are specific to certain plant species or host–pathogen systems. For example, fengycin produced by *B. subtilis* BBG111 plays an indispensable role in the induced defence state in rice (*Oryza sativa* L.) against *Rhizoctonia solani* (Chandler *et al.*, 2015). However, plipastatin (fengycin family) does not induce defence gene expression in grapevine cell suspension (Farace *et al.*, 2015). The iturin family possesses strong antifungal activity, but limited antiviral and antibacterial activity (Falardeau *et al.*, 2013). No evidence exists for iturin‐induced ISR (Falardeau *et al.*, 2013) except a recent report revealing that purified mycosubtillin (a member of the iturin family) activates defence responses in grapevine cell suspension and long‐lasting tolerance to *B. cinerea* in leaves (Farace *et al.*, 2015).

According to the literature, different *B. subtilis* strains produce different types of CLPs. For example, *B. subtilis* BBG111 synthesizes surfactin and fengycin while *B. subtilis* RFB104 produces surfactin and mycosubtilin (Chandler *et al.*, 2015). However, *B. subtilis* 916 produces surfactin, bacillomycin (iturin family), fengycin and a novel family of CLPs called locillomycin (Luo *et al.*, 2015). Therefore, different strains have different activities even against the same pathogen. For example, *B. subtilis* BBG111 but not *B. subtilis* RFB104 protects rice against *R. solani* (Chandler *et al.*, 2015). Moreover, the activity of the same strain can differ according to the plant/pathogen interaction considered. For example, *B. subtilis* BBG111 is capable of protecting rice against the necrotrophic *R. solani*, while it is ineffective against the hemibiotropic *M. oryzae* (Chandler *et al.*, 2015). It is therefore necessary to unravel the modes of action in each pathosystem and identify the active compounds for each strain, especially for those which have the potential to be used commercially.

For grapevine, some *B. subtilis* strains are used for protection against grey mould and are currently available on the market (*B. subtilis* strain QST 713, Serenade®) (Rotolo *et al.*, 2018). However, few have been developed for downy mildew. Recently, we found that the *B. subtilis* strain GLB191 (hereafter GLB191), isolated from grapevine leaves, is an efficient BCA against downy mildew in both controlled and field conditions (Zhang *et al.*, 2017). However, the mechanisms of *B. subtilis* activity against downy mildew are not clear. The deciphering of these mechanisms and further identification of the active metabolites is crucial. In the present study, we investigated the mode(s) of action of this new BCA against downy mildew with a focus on the putative role played by CLPs. Supernatants of GLB191 and mutant strains affected in CLPs synthesis were therefore produced and used. We found that GLB191 has direct effect against the pathogen and stimulates the plant defences, which results from both fengycin and surfactin secreted in the supernatant. This is the first report showing that CLPs produced by *B. subtilis* have double effects in the biotrophic pathogen *P. viticola*/grapevine pathosystem.

## Results

### *B. subtilis* GLB191 supernatant protects grapevine plants against downy mildew

**Figure 1:**
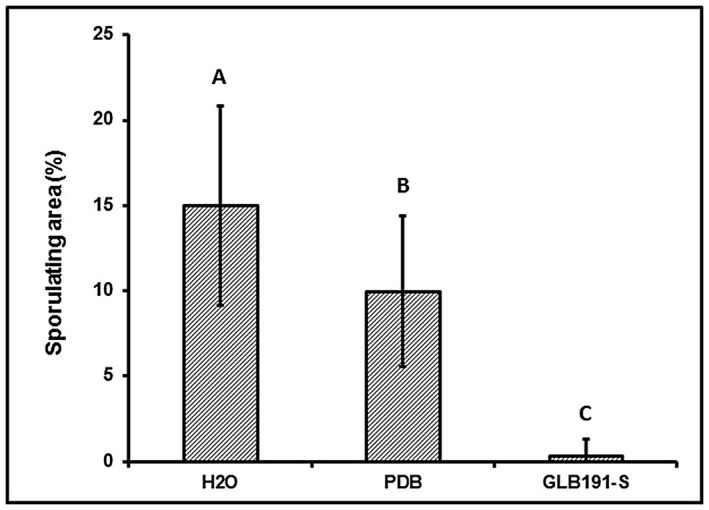
*B. subtilis* GLB191 supernatant‐induced protection of grapevine leaves against downy mildew. Sporulation of *Plasmopara viticola* was assessed with the downy mildew susceptible cultivar cv. Marselan treated with water (H_2_O), PDB medium (PDB) or the supernatant of *B. subtilis* GLB191 (GLB191‐S). Values correspond to the mean percentage obtained for 36 discs of six leaves from three plants (the second and third apical leaves from each plant). The data are representative of three independent experiments. Treatments were compared by means with the non‐parametric Kruskal–Wallis approach at the 5% significance level. Means with different letters are significantly different at *P* < 0.01 according to the Mann–Whitney pairwise post hoc test with application of the Bonferroni correction.

Foliar treatment with the supernatant of the *B. subtilis* strain GLB191 (hereafter called GLB191‐S, with S for supernatant) reduced the leaf sporulating area by 97.6%, compared to water treatment (Fig. 1). Non‐inoculated potato dextrose broth (PDB) culture medium also significantly reduced the pathogen sporulation (by 33.5% compared to the water control). However, the protective activity of GLB191‐S was markedly higher than that of the PDB medium (Fig. 1). The supernatant of GLB191 therefore plays a major role in the protection of grapevine against downy mildew.

### Direct effect of GLB191‐S on *P. viticola* zoospores

**Figure 2:**
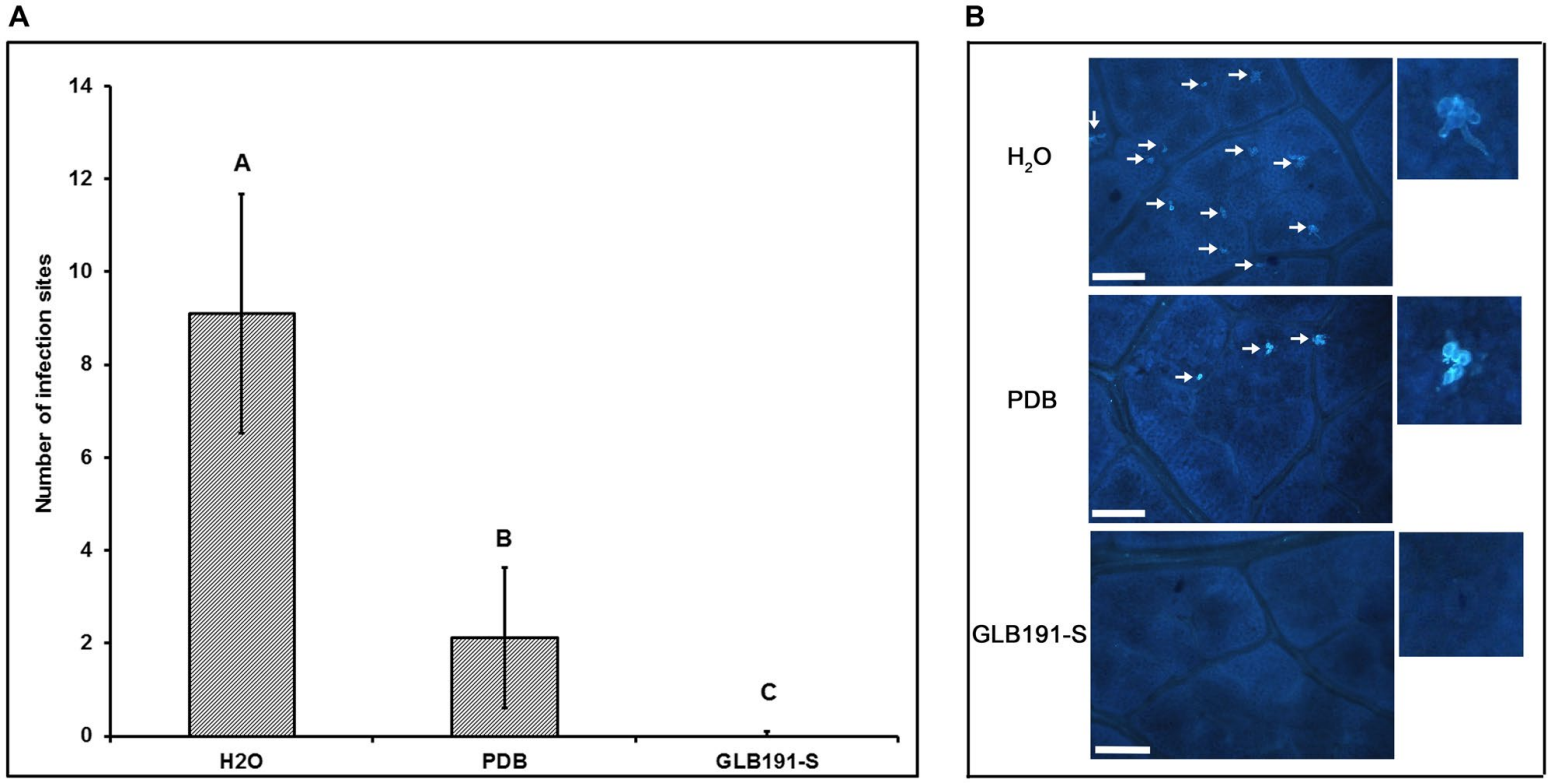
Direct effect of *B. subtilis* GLB191 supernatant on *P. viticola* zoospores. Grapevine (*V. vinifera* cv. Marselan) leaves were treated with water (H_2_O), PDB medium (PDB) or the supernatant of *B. subtilis* GLB191 (GLB191‐S) and inoculated with *P. viticola* sporangia 2 h later. At 24 h post inoculation, leaf discs were punched out from leaves and the number of infection sites (i.e. stomata with encysted zoospores of *P. viticola*) was determined by epifluorescence observations after aniline blue staining of the pathogen. Panel A: Values are the mean number of infection sites for ten discs of six leaves harvested from three plants (the second and third apical leaves from each plant). The data correspond to a representative of three independent experiments. Treatments were compared by means with the non‐parametric Kruskal–Wallis approach at the 5% significance level. Means with different letters are significantly different according to the Mann–Whitney pairwise post hoc test with application of the Bonferroni correction (*P* < 0.01). Panel B: Representative photographs of fluorescence microscopy observations. Arrows indicate the infection sites (encysted zoospores). Scale bars represent 100 μm.

In order to further unravel the mode(s) of action of GLB191 to control downy mildew, the direct activity of GLB191‐S against *P. viticola* was first determined. The number of infection sites (i.e. stomata with encysted zoospores of *P. viticola*) was determined at 24 h post inoculation (hpi) by UV epifluorescence observations after aniline blue staining of the pathogen. The highest number of infection sites (9.1 ± 2.6 per observation field) was observed for the water control (Fig. 2). PDB treatment significantly reduced their number (2.1 ± 1.5), whereas almost none could be observed on leaves treated with GLB191‐S (Fig. 2). These results indicate that GLB191‐S has a significant direct effect on zoospores.

### GLB191‐S activates callose production and defence genes in grapevine leaves

**Figure 3:**
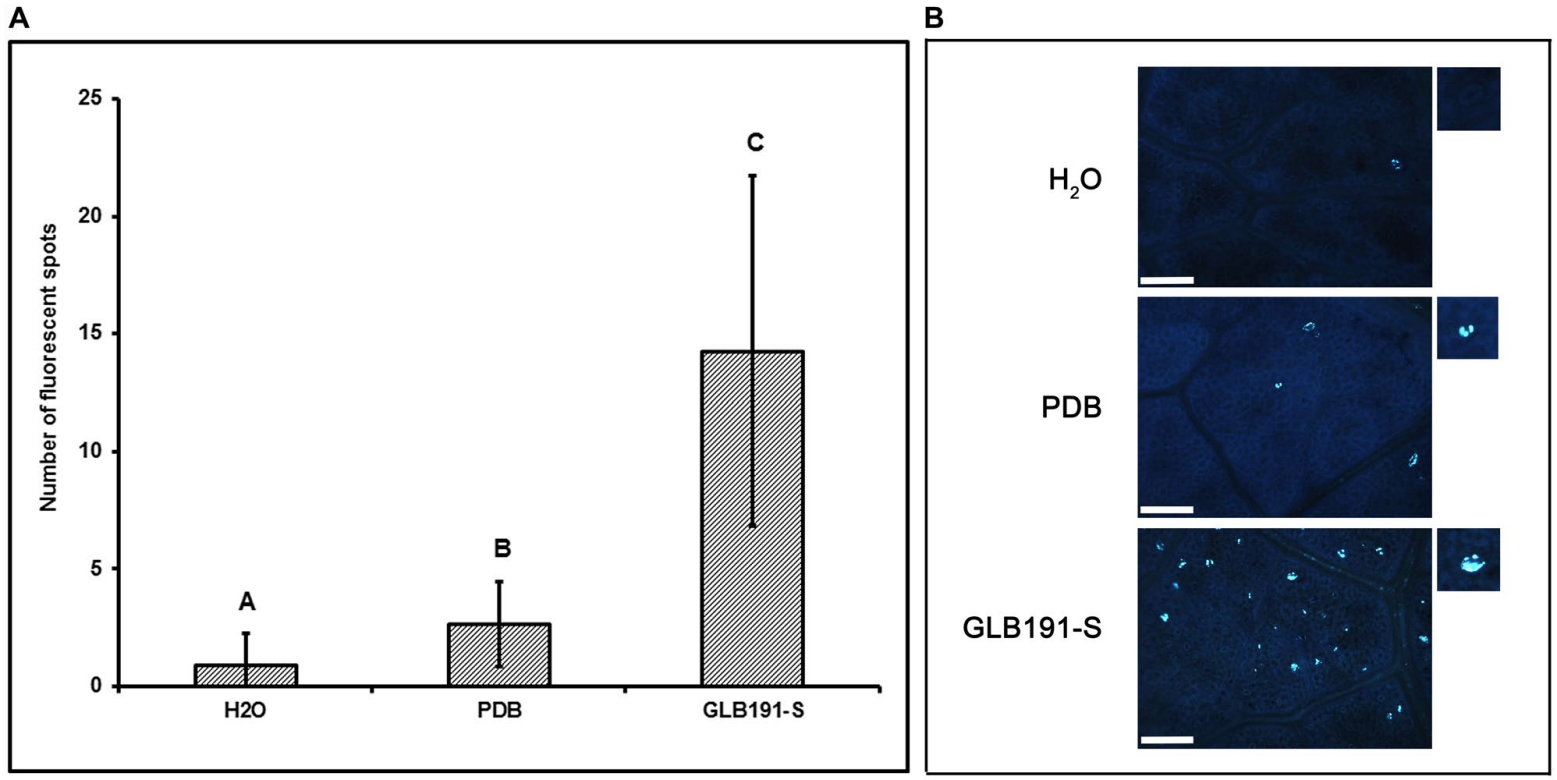
Callose production induced by *B. subtilis* GLB191 supernatant. Grapevine (*V. vinifera* cv. Marselan) leaves were treated by water (H_2_O), PDB medium (PDB) or the supernatant of *B. subtilis* GLB191 (GLB191‐S). Callose production (fluorescent spots) was assessed 3 days post treatment by epifluorescence observations after aniline blue staining. Panel A: Values are the mean number of fluorescent spots for six leaves from three plants (the second and third apical leaves from each plant). Treatments were compared by means with the non‐parametric Kruskal–Wallis approach at the 5% significance level. Means with different letters are significantly different according to the Mann–Whitney pairwise post hoc test with application of the Bonferroni correction (*P* < 0.01). The data is a representative of three independent experiments. Panel B: Representative photographs of fluorescence microscopy observations. Scale bars represent 100 μm.

Callose production was monitored by UV epifluorescence after aniline blue staining. The data revealed that the fluorescence of the spots was scarce for water‐treated leaves and slightly higher (2.7 ± 1.8) for PDB‐treated ones (Fig. 3A). In contrast, the number of spots was markedly increased after treatment by GLB191‐S (14.3 ± 7.4 per observation field) (Fig. 3A), indicating a strong callose production. Callose was localized at the level of stomata (Fig. 3B).

**Figure 4:**
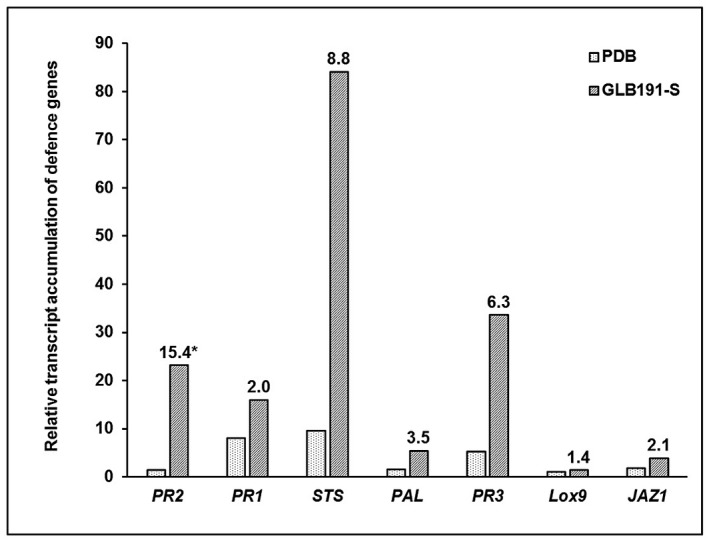
Defence‐related gene expression in grapevine leaves treated with *B. subtilis* GLB191 supernatant. The relative transcript accumulation of defence genes was determined by qRT‐PCR in grapevine (*V. vinifera* cv. Marselan) leaves 24 h post treatment with water (H_2_O), PDB medium (PDB) or the supernatant of *B. subtilis* GLB191 (GLB191‐S). Results represent relative fold expression calculated with the 2^−ΔΔCt ^method, compared to the reference gene *EF1γ* and to the water control. The data are representative of three independent experiments. *PR2*, PR protein 2 β‐1,3‐glucanase; *PR1*, PR protein 1; *STS*, stilbene synthase; *PAL*, phenylalanine ammonia lyase; *PR3*, PR protein 3 chitinase 4c; *Lox9*, lipoxygenase 9; *JAZ1*, jasmonate ZIM‐domain protein. *The values above each column indicate the relative fold of transcript accumulation in leaves treated with GLB191‐S compared to that of PDB‐treated ones.

Quantitative reverse transcription‐polymerase chain reaction (qRT‐PCR) was next used to analyse the expression of a set of defence genes known to be involved in different defence pathways in grapevine: *PAL* (coding for phenylalanine ammonia lyase, a key enzyme of the phenylpropanoid pathway), *STS* (encoding stilbene synthase, downstream of PAL and responsible for the synthesis of resveratrol, the main phytoalexin produced by grapevine in response to biotic or abiotic stresses), *PR1* (encoding the pathogenesis‐related (PR) protein 1 that is considered as a marker of the salicylic acid signalling pathway), *PR2* (encoding the PR protein 2 β‐1,3‐glucanase), *PR3* (encoding the PR protein 3 chitinase 4c), *Lox9* (encoding the lipoxygenase 9, an enzyme involved in jasmonic acid signalling) and *JAZ1* (encoding the jasmonate ZIM‐domain protein 1, another marker of the jasmonic acid signalling pathway). In response to GLB191‐S treatment, the expression of all genes tested was induced compared to the water control, except for *Lox9*, with difference of intensity (Fig. 4). *STS* was the most up‐regulated gene and *JAZ1* the lowest one, with around 80‐ and 4‐fold relative expression to water, respectively. PDB medium also induced the expression of some defence genes, especially *PR1*, *STS* and *PR3*. However, the transcript accumulation of these defence genes was always lower in response to PDB treatment than that found in response to GLB191‐S. Moreover, results showed that *PR2*, *STS* and *PR3* were the most up‐regulated genes by GLB191‐S with 15.4‐, 8.8‐ and 6.3‐fold relative to PDB, respectively.

Altogether these results demonstrate that GLB191‐S stimulates various grapevine defence responses.

### Both surfactin and fengycin contribute to GLB191 activity against downy mildew

In order to investigate whether CLPs contribute to the protection of GLB191‐S, deletion mutants affected in the synthesis of fengycin (∆*ppsB*), surfactin (∆*srfAA*) or both (∆*ppsBsrfAA*) were generated. First, the CLP concentration in the supernatant of GLB191 and its mutants affected in CLP production was determined by high‐performance thin‐layer chromatography (HPTLC) analysis. As expected, GLB191 produces fengycin and surfactin (56.2 and 52.1 mg/L, respectively) (Table 1). Deletion of *srfAA* resulted in no surfactin production and a level of fengycin similar to those of GLB191 (Table 1). Deletion of *ppsB* resulted in no fengycin production as expected but in a reduced surfactin production (22.5 mg/L) (Table 1). None of the analysed CLPs could be detected in the supernatant of the double mutant and no iturin A was detected in all strain supernatants tested (Table 1).

**Table 1:** Production of cyclic lipopeptides (CLPs) by *B. subtilis* GLB191 and its derived mutants grown in PDB medium.*

	Strain			
CLPs	GLB191	Δ*ppsB*	Δ*srfAA*	Δ*ppsBsrfAA*
Surfactin	52.1 ± 8.2†	22.5 ± 12.6	0.0 ± 0.0	0.0 ± 0.0
Fengycin	56.2 ± 3.8	0.0 ± 0.0	54.6 ± 3.1	0.0 ± 0.0
Iturin A	0.0 ± 0.0	0.0 ± 0.0	0.0 ± 0.0	0.0 ± 0.0

* The values are means from three experiments.

† Mean ± SD, mg/L.

**Figure 5:**
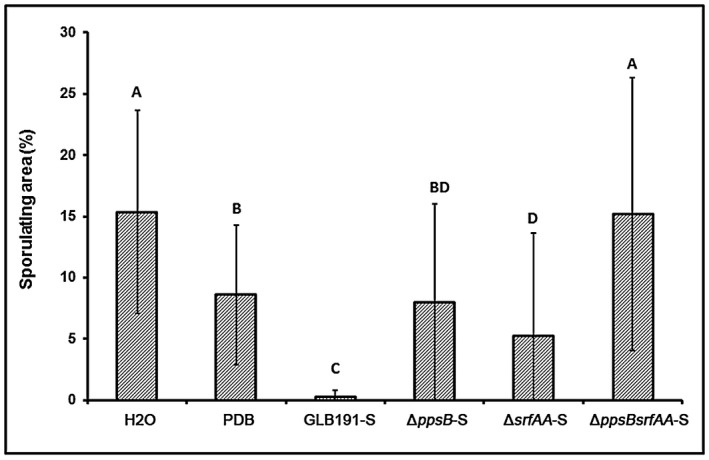
Protection of grapevine leaves against downy mildew induced by *B. subtilis* GLB191 and its derived mutants. Sporulation of *Plasmopara viticola* was assessed with the downy mildew susceptible cultivar cv. Marselan treated with water (H_2_O), PDB medium (PDB) or the supernatant of the wild‐type *B. subtilis* GLB191 (GLB191‐S) and its mutants Δ*ppsB* (Δ*ppsB*‐S), Δ*srfAA* (Δ*srfAA*‐S), Δ*ppsBsrfAA* (Δ*ppsBsrfAA*‐S). Values correspond to the mean percentage obtained for 36 discs of six leaves from three plants (the second and third apical leaves from each plant). The data are the mean of three independent experiments. Treatments were compared by means with the non‐parametric Kruskal–Wallis approach at the 5% significance level. Means with different letters are significantly different according to the Mann–Whitney pairwise post hoc test with application of the Bonferroni correction (*P* < 0.01).

We then investigated the protection efficacy of the supernatants of GLB191 mutants against downy mildew. Foliar treatment of Marselan with the supernatant of the single mutant Δ*ppsB* (Δ*ppsB*‐S) or Δ*srfAA* (Δ*srfAA*‐S) resulted in a significant increase of *P. viticola* sporulation compared to GLB191‐S (Fig. 5). Treatment with the supernatant of the double mutant Δ*ppsBsrfAA* (Δ*ppsBsrfAA*‐S) showed a significantly higher sporulation of *P. viticola* compared to the single mutants. It did not induce protection, as indicated by a level of sporulation similar to that of the water control (Fig. 5). A lower level of sporulation was observed in response to PDB treatment compared to the water control (as in Fig. 1) and with *ΔppsBsrfAA*‐S treatment.

### Both surfactin and fengycin contribute to the direct effect of GLB191 on *P. viticola* zoospores

**Figure 6:**
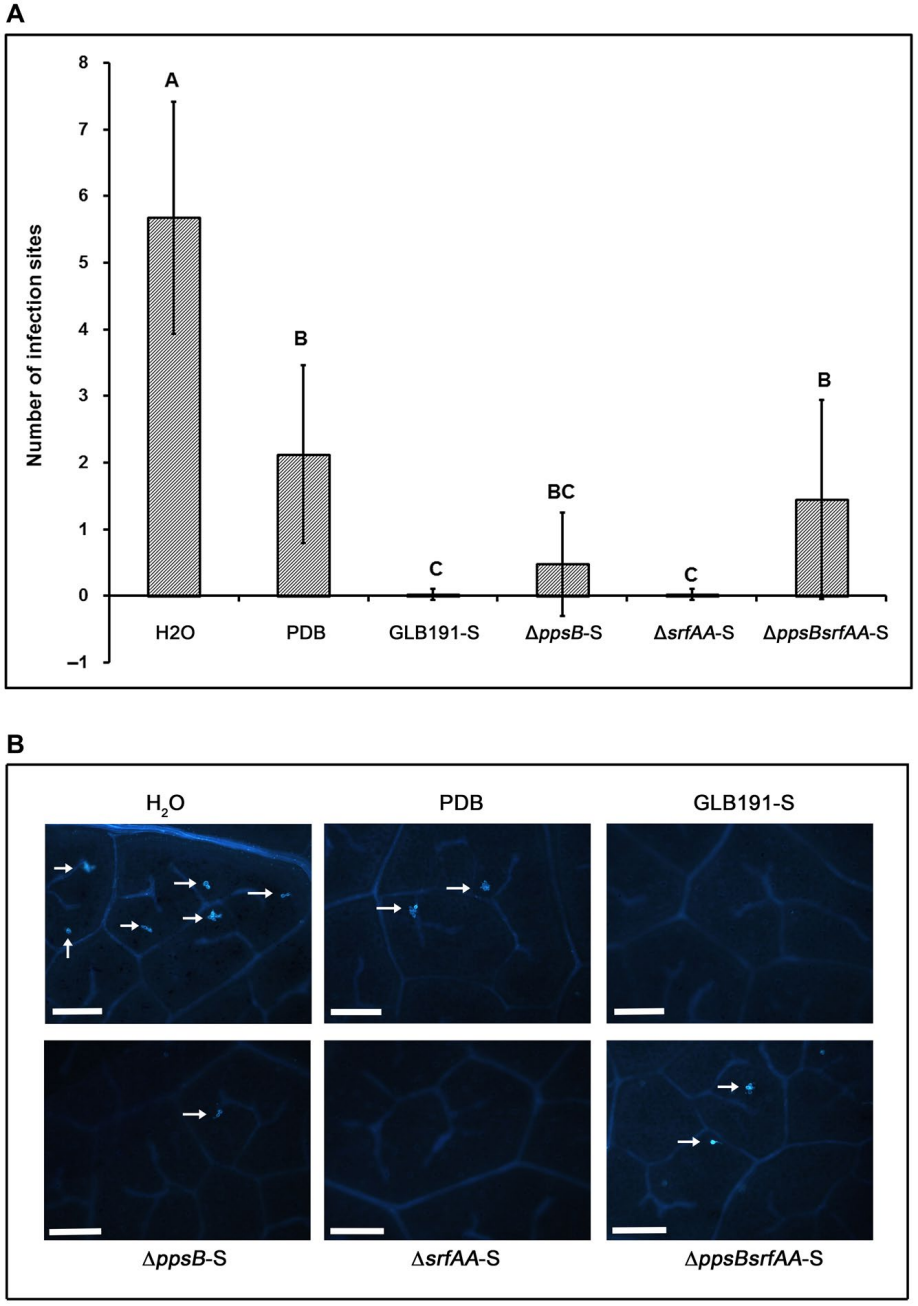
Direct effect of the supernatants of *B. subtilis* GLB191 and its derived mutants on *P. viticola* zoospores. Grapevine (*V. vinifera* cv. Marselan) leaves were treated with water (H_2_O), PDB medium (PDB) or the supernatant of *B. subtilis* GLB191 (GLB191‐S) and its mutants Δ*ppsB* (Δ*ppsB*‐S), Δ*srfAA* (Δ*srfAA*‐S), Δ*ppsBsrfAA* (Δ*ppsBsrfAA*‐S), and inoculated with *P. viticola* sporangia 2 h later. At 24 hpi, leaf discs were punched out from leaves and the number of infection sites (i.e. stomata with encysted zoospores of *P. viticola*) was determined by UV epifluorescence observations after aniline blue staining of the pathogen. Panel A: Values are the mean number of infection sites for ten discs of six leaves harvested from three cuttings (the second and third apical leaves from each plant). The data correspond to a representative of three independent experiments. Treatments were compared by means with the non‐parametric Kruskal–Wallis approach at the 5% significance level. Means with different letters are significantly different according to the Mann–Whitney pairwise post hoc test with application of the Bonferroni correction (*P* < 0.05). Panel B: Representative photographs of fluorescence microscopy observations. Arrows indicate the infection sites (encysted zoospores). Scale bars represent 100 μm.

The supernatants of the different strains were next compared regarding their effects against *P. viticola* infection. Infection sites on leaves treated with GLB191‐S and *ΔsrfAA*‐S were scarce (Fig. 6). They seemed slightly more abundant in leaves treated with Δ*ppsB*‐S but no statistically significant difference was found compared to GLB191‐S and *ΔsrfAA*‐S. Deletion of both *ppsB* and *srfAA* (Δ*ppsBsrfAA*‐S) resulted in a significantly higher number of infection sites compared to the wild type and a similar level to the PDB medium (Fig. 6). These results indicate that both surfactin and fengycin contribute to the direct effect of GLB191‐S on *P. viticola* zoospores.

### Both surfactin and fengycin contribute to the stimulation of grapevine defences

**Figure 7:**
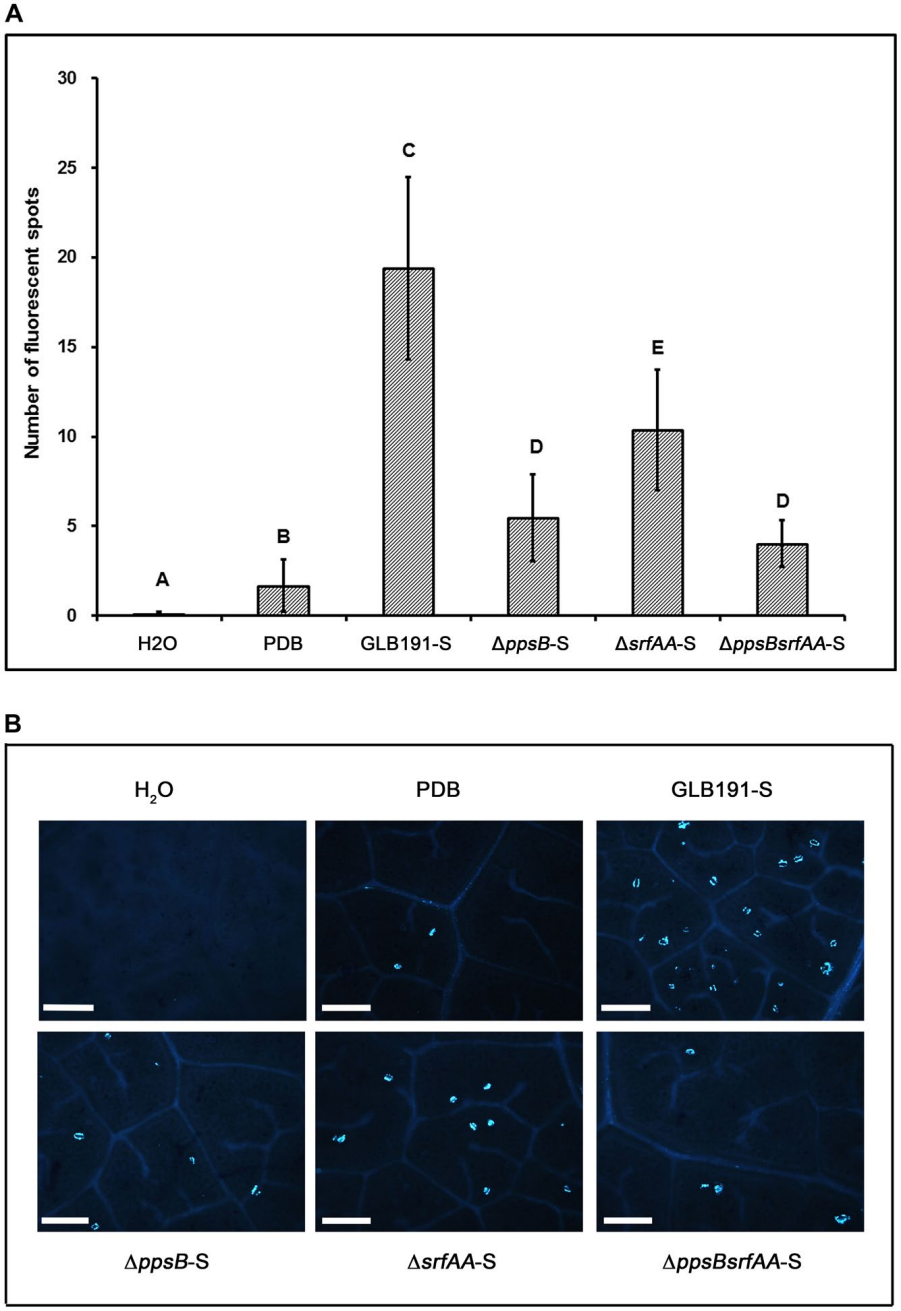
Callose production induced by the supernatant of *B. subtilis* GLB191 and its derived mutants. Callose deposition in grapevine (*V. vinifera* cv. Marselan) leaves treated by water (H_2_O), PDB medium (PDB) or the supernatant of the wild‐type *B. subtilis* GLB191 (GLB191‐S) and its mutants Δ*ppsB* (Δ*ppsB*‐S), Δ*srfAA* (Δ*srfAA*‐S), Δ*ppsBsrfAA* (Δ*ppsBsrfAA*‐S) was assessed 3 days post treatment by epifluorescence observations after aniline blue staining. Panel A: Values are the mean estimation for leaves from three plants (the second and third apical leaves from each plant) in the same treatment. Treatments were compared by means with the non‐parametric Kruskal–Wallis approach at the 5% significance level. Means with different letters are significantly different according to the Mann–Whitney pairwise post hoc test with application of the Bonferroni correction (*P* < 0.05). The data are representative of three independent experiments. Panel B: Representative photographs observed using a fluorescence microscope. Scale bars represent 100 μm.

We further investigated whether the supernatants of GLB191 mutants were still efficient to induce grapevine defences. The data showed that callose production in Δ*ppsB‐*S‐ or Δ*srfAA*‐S‐treated plants was significantly reduced compared to that in GLB191‐S‐treated plants but significantly higher than that of the PDB and water control (Fig. 7). Moreover, callose production in Δ*ppsBsrfAA*‐S‐treated leaves was similar to that of Δ*ppsB‐*S‐treated ones and significantly higher than that of the PDB control (Fig. 7). These results indicate that both surfactin and fengycin are main factors in the supernatant of GLB191 causing callose production in grapevine leaves.

**Figure 8:**
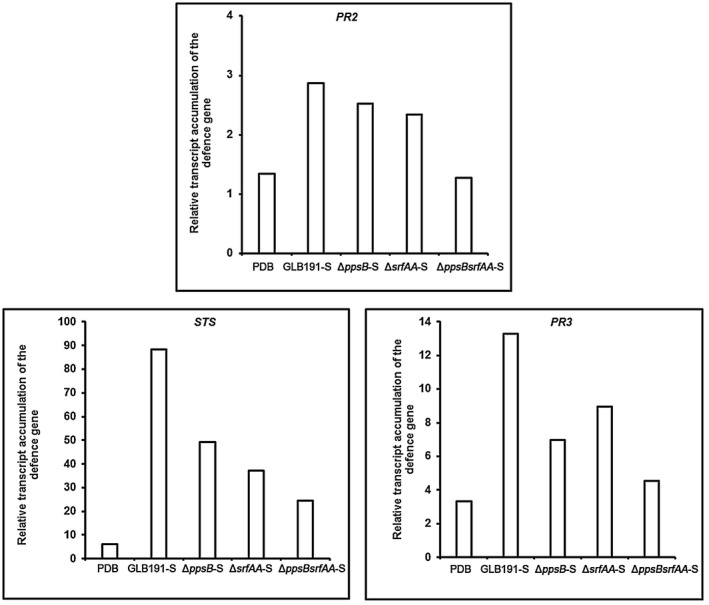
Defence‐related genes expression in grapevine leaves treated with supernatant of *B. subtilis* GLB191 and its derived mutants. The relative transcript accumulations of genes *PR2* (encoding the pathogenesis‐related protein 2 β‐1,3‐glucanase), *STS* (encoding stilbene synthase) and *PR3* (encoding the pathogenesis‐related protein 3 chitinase 4c) were determined by qRT‐PCR in leaves treated with water, PDB medium (PDB) or the supernatant of *B. subtilis* GLB191 (GLB191‐S) and its mutants Δ*ppsB* (Δ*ppsB*‐S), Δ*srfAA* (Δ*srfAA*‐S), Δ*ppsBsrfAA* (Δ*ppsBsrfAA*‐S). Results represent relative fold expression calculated with the 2^−ΔΔCt ^method, compared to the reference gene *EF1γ* and to the water control. The data are representative of three independent experiments.

For defence gene expression, we focused on *PR2*, *PR3* and *STS* as they were highly induced by GLB191‐S. The qRT‐PCR data revealed that deletion of both *ppsB* and *srfAA* (Δ*ppsBsrfAA*‐S) resulted in a stronger reduction of transcript accumulation of these defence genes than that observed for single mutants *ppsB* or *srfAA* (Δ*ppsB*‐S or Δ*srfAA*‐S) compared to the wild type (GLB191‐S) (Fig. 8). These results indicate again that both surfactin and fengycin contribute to the induction of defence gene expression after GLB191‐S treatment.

## Discussion

We have previously observed that the endophytic bacterial strain *B. subtilis* GLB191 isolated from grapevine leaves has a strong preventive activity against *P. viticola* on the susceptible cultivars *V. vinifera* L. cv. Muscat Hamburg (a table grape cultivar) and cv. Cabernet Sauvignon (a wine grape cultivar) in both leaf disk assays and the field. Assays were performed with the culture broth containing both bacterial cells and their metabolites (Zhang *et al.*, 2017). As *B. subtilis* is known to produce biologically active molecules, especially CLPs (Ongena and Jacques, 2008; Stein, 2005), experiments were performed in order to determine if these molecules were involved in GLB191 activity against *P. viticola*.

First, we demonstrated that the supernatant of GLB191, which is likely to contain CLPs, was also active against downy mildew. Preliminary experiments were performed with Luria‐Bertani (LB) and Landy media generally used for *B. subtilis* cultivation as they favour the production of CLPs (Fan *et al.*, 2017a; Landy *et al.*, 1948; Luo *et al.*, 2015). However, their protective effect on *P. viticola* was stronger compared to PDB medium (data not shown). We therefore decided to use PDB as the culture medium for this study to reduce the effect of the medium itself.

The modes of action of GLB191‐S were next investigated. *B. subtilis* has been reported to protect plants against various phytopathogens by stimulating the plant host defences. Moreover, some CLPs are considered to be a new class of microbe‐associated molecular patterns (MAMPs) (Ongena and Jacques, 2008), i.e. as elicitors of plant immunity (Boutrot and Zipfel, 2017). GLB191‐S was therefore tested as putative defence inducer. In the present study, we showed that GLB191‐S induces callose production (Fig. 3A). In grapevine, this defence event was previously reported in response to several defence elicitors such as sulphated laminarin (PS3) or β‐aminobutyric acid (BABA), and was clearly associated with induced resistance against *P. viticola* (Hamiduzzaman *et al.*, 2005; Trouvelot *et al.*, 2008; Yu *et al.*, 2016). Callose production is also involved in the natural resistance of grapevine species and hybrids to *P. viticola*. For example, Gindro *et al.*, (2003) have reported callose production in leaf stomata of the downy mildew tolerant hybrid Solaris after challenge with *P. viticola*. Similarly, callose was localized at the level of stomata of GLB191‐S‐treated leaves (Fig. 3B), suggesting a role in the prevention of infection (Gindro *et al.*, 2003; Trouvelot *et al.*, 2008). GLB191‐S also induced the expression of several defence genes (Fig. 4), suggesting that different defence responses are activated. However, markers of SA‐ and JA‐signalling pathways were weakly induced (Fig. 4), suggesting a local induced resistance rather than an ISR. However, this hypothesis would need to be confirmed by further experiments.

GLB191‐S therefore induces grapevine resistance against downy mildew by both direct effect against the pathogen (we have shown a reduction in the number of infection sites) and plant defence stimulation. Since CLPs of *B. subtilis* are particularly important in plant protection (Stein, 2005), we next investigated whether they contribute to the activity of GLB191‐S. Deletion mutants affected in the synthesis of fengycin (∆*ppsB*), surfactin (∆*srfAA*) or both (∆*ppsBsrfAA*) were generated. Although iturin is known to display strong antifungal activity (Maget‐Dana and Peypoux, 1994), a search of iturin‐related genes in the GLB191 genome surprisingly yielded zero results. Thus, a mutant for iturin was not constructed. The CLP concentration in the supernatant of GLB191 and its mutants was determined. The absence of iturin A (Table 1) is consistent with the genome search that could not allowed us to identify an iturin‐related gene cluster. Deletion of *ppsB* resulted in no fengycin production and a reduced surfactin production (Table 1), which is contrary to previous reports (Coutte *et al.*, 2010; Luo *et al.*, 2015). In *B. subtilis* 916, deletion of *fenA*, which is involved in fengycin synthesis, resulted in increased surfactin production (Luo *et al.*, 2015). Coutte *et al.* (2010) also observed an enhanced surfactin production after disruption of the plipastatin operon in *B. subtilis* 168 derivative BBG113, indicating interdependency of surfactin and plipastatin biosynthesis. This phenomenon could not be observed in the *ppsB* mutant of GLB191. The lower production of surfactin in ∆*ppsB* revealed in this study is surprising and would merit further investigation. It might be due to a specific crosstalk in the regulation of lipopeptide production in the GLB191 strain.

Using mutants, we observed that the protective activity against downy mildew was reduced in the absence of either surfactin or fengycin and it was lost in the absence of both of them (Fig. 5), indicating that both fengycin and surfactin are the main active compounds of GLB191‐S against *P. viticola*. Farace *et al.* (2015) observed that only surfactin and mycosubtilin (iturin family), but not plipastatin (i.e. fengycin), enhanced tolerance of grapevine leaves against the necrotrophic fungus *B. cinerea*. It therefore seems that the relative importance of these three CLPs families to induce resistance in grapevine could be pathogen dependent. The lower level of sporulation observed for the PDB medium treatment compared to the water control and Δ*ppsBsrfAA* (Fig. 5) indicates that some components in the PDB medium have a protective activity against *P. viticola* and could probably be used by GLB191 bacteria for growth.

Surfactin is known to have a strong direct antibacterial activity and stimulate plant defences whereas fengycin has a strong direct antifungal effect and stimulates defence in certain plant species or specific host–pathogen systems (Ongena and Jacques, 2008). Therefore, reduction of *P. viticola* sporulation may be due to both direct antimicrobial effect and/or induced resistance resulting from surfactin and/or fengycin.

The direct antimicrobial effect of CLPs on phytopathogens is due to the interaction with the plasma membrane of the pathogen. CLPs can cause pores to form on the membrane, causing an osmotic imbalance that ultimately results in cell death (Pérez‐García *et al.*, 2011). According to the literature, surfactin shows antiviral and antibacterial activity whereas fengycin has strong activity against fungi, especially filamentous ones (Vanittanakom *et al.*, 1986). The differential activities of CLP families are linked to their respective mechanisms of action on phytopathogen biological members (Falardeau *et al.*, 2013). Moreover, the antimicrobial activity of surfactin and iturin is dose‐dependent whereas it is all or none for fengycin (Falardeau *et al.*, 2013). In our study, Δ*ppsB*‐S, which produces surfactin but no fengycin (Table 1), and Δ*srfAA*, which produces fengycin but no surfactin (Table 1), showed direct effect against *P. viticola* infection (Fig. 6), suggesting that both fengycin and surfactin have effect against this oomycete. There was no statistically significant difference observed in the number of infection sites on leaves treated with GLB191‐S and the single mutant Δ*ppsB*‐S or Δ*srfAA*‐S. The slightly higher and more variable number of infection sites in response to Δ*ppsB*‐S might be due to the half concentration of surfactin present in this supermatant (Table 1). The double mutant Δ*ppsBsrfAA*, which produces no fengycin and surfactin (Table 1), lost the inhibition of *P. viticola* zoospores (Fig. 6). These results suggest that the concentration of surfactin in Δ*ppsB*‐S or fengycin in Δ*srfAA*‐S is enough to inhibit zoospores of *P. viticola*.

The analysis of callose production and defence gene expression showed that surfactin and fengycin are the main factors in the supernatant of GLB191 responsible for stimulation of plant defences. However, it seems that the different defence genes are not regulated in a similar manner by the two CLPs. Previous studies have reported the role of surfactin in the activation of plant defences and suggested that it could result from its insertion into the plant plasma membrane (Henry *et al.*, 2011). Using cell suspensions, Farace *et al.* (2015) have shown that purified surfactin, mycosubtillin and plipastatin differently activate defence responses in grapevine. In their conditions, surfactin and mycosubtilin stimulated grapevine innate immune responses whereas plipastatin perception only resulted in early signalling activation. Mycosubtilin activated the strongest gene expression, but it was associated with cell death.

Altogether, this study demonstrates that both surfactin and fengycin in the supernatant contribute to the protection of a natural strain GLB191 against downy mildew which resulted from both their direct anti‐oomycete activity against *P. viticola* and stimulation of the plant defences. However, different *Bacillus* strains are known to produce variable concentrations of each CLP in natural conditions when they interact with the plant at different times for ecological fitness (Debois *et al.*, 2015). Further research is needed to determine which CLP is involved at a given time and the concentrations of each CLP when GLB191 is sprayed on the leaves of grapevine.

## Experimental procedures

### Plant material

The grapevine cultivar* Vitis vinifera* cv. Marselan (Cabernet sauvignon × Grenache), susceptible to *P. viticola*, was used in this study. Plants were grown in a glasshouse as described previously (Krzyzaniak *et al.*, 2018). In brief, plants were produced from herbaceous cuttings planted in individual pots at 23 and 18 °C (day and night, respectively) with a photoperiod of 16 h of light until they developed six to eight leaves. Plants were watered daily and fertilized once a week (N/P/K 10‐10‐10, Plantin, Courthezon, France). The second and third youngest fully expanded leaves were used for experiments.

### Bacterial strains and cultural conditions

The bacterial strains and plasmids used in this study are listed in Table 2. *B. subtilis* strains were stored at −80 °C in 15% glycerol. For experiments, they were grown in Potato Dextrose Broth (PDB) (Conda SA, Madrid, Spain) medium at 37 °C. *E. coli* strains were grown in LB at 37 °C. Antibiotics were added at the following concentrations when required: ampicillin (Ap, 100 μg/mL), erythromycin (Em, 5 μg/mL).

**Table 2:** Strains and plasmids used in this study.

Strains or plasmids	Characteristics^a^	Sources or references
**Strains**		
***B. subtilis***		
GLB191	Wild‐type strain, isolated from grapevine leaves	Zhang *et al.* (2017)
Δ*ppsB*	*ppsB* deletion mutant of GLB191, markerless	This work
Δ*srfAA*	*srfAA* deletion mutant of GLB191, markerless	This work
Δ*ppsBsrfAA*	*ppsB* and *srfAA* double mutant of GLB191, markerless	This work
***E. coli***		
DH5α	F‐φ80 *lac* ZΔM15 Δ(*lacZYA‐*arg *F*) *U*169 *endA*1 *recA*1 *hsdR*17(rk‐,mk+) *supE*44λ‐*thi* ‐1 *gyrA*96 *relA*1 *phoA*	Life Technologies
EC135	EC132 Δ*dam*::*FRT*, genotype of R‐M systems: *mcrA* Δ(*mrr*‐*hsdRMS*‐*mcrBC*) Δ*dcm*::*FRT* Δ*dam*::*FRT*	Zhang *et al.* (2012)
**Plasmids**		
pMAD	Shuttle vector for allele replacement; Amp^R^ (*E. coli*), Em^R ^(*Bacillus*); containing* bgaB* gene encoding a thermostable β‐galactosidase	Arnaud *et al.* (2004)
pMAD‐*ppsB*	A fusion of upstream and downstream of* ppsB* cloned into pMAD for allele replacement; Amp^R^ (*E. coli*), Em^R ^(*Bacillus*)	This work
pMAD‐*srfAA*	A fusion of upstream and downstream of* srfAA* cloned into pMAD for allele replacement; Amp^R^ (*E. coli*), Em^R ^(*Bacillus*)	This work

### Mutant construction

Markerless deletion mutants were constructed using the temperature‐sensitive suicide plasmid pMAD as described previously (Fan *et al.*, 2017a). Upstream and downstream regions of the target gene (named X hereafter) were amplified from the genomic DNA of GLB191 using the primer pairs X‐up‐F/X‐up‐R and X‐dn‐F/X‐dn‐R, respectively. The two DNA fragments were joined together by PCR amplification using the primers X‐up‐F and X‐dn‐R. The resulting fragment was digested and cloned into pMAD, generating pMAD‐X in *E. coli* DH5α. The pMAD‐X plasmid was purified from *E. coli* DH5α and mobilized into *E.coli* EC135 by heat shock and then into GLB191 by electroporation. Erythromycin resistant (Em^R^) and blue transformants were obtained after incubation at 30 °C for 2 days on LB plates containing Em and X‐Gal (40 μg/mL), followed by incubation in LB broth containing Em at 42 °C with shaking at 180 r min^−1^ for 8–10 h for the first allelic exchange. Em^R^ and blue transformants were obtained from LB plates supplemented with Em and X‐Gal and then incubated in LB broth at 25 °C with shaking at 180 r min^−1^ for 24 h for the second allelic exchange. Em‐sensitive and white clones were isolated and confirmed by PCR with primers X‐Up‐F and X‐Dn‐R and subsequently by sequencing. The primers used are listed in Table S1.

### Preparation and application of the supernatant of *B. subtilis* GLB191 and its derivatives

Bacteria were grown in PDB medium at 37 °C with shaking at 180 r min^−1^ for 48 h. Cell‐free supernatants were obtained after centrifugation at 6000 × *g* for 20 min at 4 °C, and then filtration using a 0.22‐μm pore size filter. The supernatants were applied to leaves until run‐off using a manual sprayer. Plants sprayed with sterilized distilled H_2_O and the PDB medium, respectively, were used as controls.

### *P. viticola* preparation

The *P. viticola* isolate used for this study was maintained on Marselan plants in the glasshouse as previously described (Trouvelot *et al.*, 2008). To obtain sporangia, plants presenting oily spot symptoms were placed in the dark at > 95% relative humidity (RH) overnight to induce sporulation. Sporangia were then collected from the lower side of leaf using a brush and suspended in distilled water. The concentration was adjusted to 2.10^4^ or 10^5 ^sporangia per milliliter (depending on experiments) using a haemocytometer.

### Protection assays

Two days post‐treatment (dpt) with H_2_O, PDB or supernatant(s), the lower face of leaves was inoculated with a freshly prepared sporangia suspension (2.10^4^ sporangia per milliliter) using a manual sprayer. Inoculated plants were then placed overnight in a humid chamber (RH > 95%) and then moved back to the glasshouse and grown as described above. Six days post‐inoculation, six discs were punched from each leaf and then placed on wet paper filter in a humid chamber overnight to provoke pathogen sporulation. Disease severity was then assessed by measuring the leaf area covered by sporulation using the image analysis Visilog 6.9 software (Noesis, Paris, France) (Kim Khiook *et al.*, 2013). Three independent biological repeats were conducted.

### Evaluation of the direct effect on *P. viticola*

The lower face of leaves which were treated with H_2_O, PDB or supernatant(s) was inoculated with a freshly prepared sporangia suspension (2.10^5^ sporangia per milliliter) 2 h post treatment (hpt). This time is sufficient to highlight putative activity against *P. viticola* and too short to activate defence responses (Krzyzaniak *et al.*, 2018). Leaves were harvested 24 h post inoculation (hpi) and discs (0.7 cm in diameter) were punched from each leaf (ten discs per leaf). They were subsequently bleached first with pure methanol at least 2 days and then chloral hydrate solution (1.0 g/L) for 12–24 h until they become completely transparent. Infection sites (corresponding to encysted zoospores) were detected after aniline blue staining (Gauthier *et al.*, 2014). Four representative fields of each disc and ten discs per condition were observed by epifluorescence microscopy [magnification ×200, λexc = 340–380 nm, λem = 425 nm (long pass filter)]. Three independent biological repeats were conducted.

### Quantification of callose deposition

Leaves were treated with H_2_O, PDB or supernatant(s) and harvested 3 dpt. Ten discs per leaf (0.7 cm in diameter) were punched. Callose deposition was revealed after tissue clearance and aniline blue staining as describe above. The number of deposits (fluorescent spots) was determined by epifluorescence microscopy observations. Three independent biological repeats were conducted.

### RNA extraction and reverse transcription

The second‐youngest fully expanded leaves of plants (four plants/condition) were treated with H_2_O, PDB or supernatants and harvested at 24 hpt and pooled. They were ground in liquid nitrogen and total RNA was isolated using PureLink^®^ Plant RNA Reagent (Invitrogen, Thermofisher, Carlsbad, USA) according to the manufacturer's instructions with minor modification. In brief, approximately 50 mg of leaf powder were incubated with 0.5 mL of Plant RNA Purification Reagent for 5 min at room temperature. After centrifugation at 12 000 × *g* for 2 min, the supernatant was transferred to a clean RNase‐free tube and equal volume of chloroform/IAA (24:1) was added to the sample. After thoroughly shaking, samples were centrifuged for 10 min at 12 000 × *g* at 4 °C and the upper aqueous phase was transferred to a clean RNase‐free tube. An equal volume of isopropanol and 40% volume of 5 M NaCl were then added to the sample. After incubation for 10 min at room temperature, samples were centrifuged for 10 min at 12 000 × *g* at 4 °C. The supernatant was removed and the pellet was washed with 1 mL of 75% ethanol. After centrifugation at 12 000 × *g* for 1 min, the supernatant was removed and the pellet was air dried and then suspended in 30 μL of distilled water. Then the RNA was treated with DNAse using DNA‐free^TM^ kit DNAse treatment and removal (Invitrogen, Thermofisher, Carlsbad, USA). The concentration of RNA extracts was determined by spectrophotometry. RNAs were reverse‐transcribed using the Superscript III Reverse Transcriptase kit (Invitrogen, Life Technologies, Saint Aubin, France), random hexamers, 1 mg of DNA‐free total RNA and anchored oligo‐dT 3:1 as primers according to the manufacturer's instructions.

### Quantitative RT‐PCR

qRT**‐**PCR was used to measure transcript levels of target genes with the primers listed in Table S2. The qRT**‐**PCR experiments were performed with the ABsolute^TM^ SYBRGreen Low ROX qPCR Mix (Thermo Scientific, Waltham, MA, USA) in a LightCycler480 (Roche Applied Science, Penzberg, Germany) using a thermal cycling profile of 95 °C 15 min; 40 cycles of 95 °C for 15 s, 60 °C for 30 s and 72 °C for 45 s. The melting curve of each reaction was produced to ensure a single amplicon. The absence of primer‐dimer formation was checked in no‐template controls. The mean cycle threshold (Ct) value of a sample's technical triplicates was used for further analysis. Relative gene expression was determined with the formula fold induction = 2^−∆∆Ct^ (Livak and Schmittgen, 2001) where ∆∆Ct = ∆Ct (treated sample) – ∆Ct (control, i.e. water‐treated sample) and ∆Ct = Ct (target gene) – Ct (reference gene). *EF1γ* encoding the elongation factor 1 gamma was used as reference gene. Three independent biological repeats were conducted.

### Quantification of cyclic lipopeptides

Supernatants of GLB191 and its derivatives were prepared as described above. Lipopeptides were quantified by HPTLC as previously described (Geissler *et al.*, 2017). In brief, 2 mL cell‐free broth was extracted threefold with each 2 mL chloroform/methanol 2:1 (v/v). The solvent layers obtained after each extraction were pooled and evaporated to dryness in a rotary evaporator (RVC2‐25 Cdplus, Martin Christ Gefriertrocknungsanlagen GmbH, Osterode am Harz, Germany) at 10 mbar and 40 °C. For HPTLC analysis, samples were resuspended in 2 mL methanol and applied as 6 mm bands on HPTLC silica gel 60 plates from Merck (Darmstadt, Germany). A standard containing the cyclic lipopeptides surfactin, iturin A (both Sigma‐Aldrich Laborchemikalien GmbH, Seelze, Germany) and fengycin (Lipofabrik, Villeneuve d'Ascq, France) with a concentration of 0.1 mg/mL each was applied in a range from 30 ng/band to 600 ng/band. The first development was conducted using chloroform/methanol/water (65:25:4, v/v/v) and the second development using butanol/ethanol/0.1% acetic acid (1:4:1, v/v/v), both over a migration distance of 60 mm. After each development, the plate was scanned at 195 nm, and surfactin and iturin A were evaluated after the first and fengycin after the second development. Three independent biological repeats were conducted.

### Statistical analysis

Treatments were compared by means of non‐parametric Kruskal–Wallis approaches at the 5% significance level. Data sets were grouped according to the Mann–Whitney pairwise post hoc test with application of Bonferroni correction. Statistical significance is acknowledged between two conditions if they have no letter (A, B, C etc.) in common.

## Supporting information


**Table S1** Primers used for mutant construction.

**Table S2** Primers used for qRT PCR.
