# Supplementary material for: Surfactin and fengycin contribute to the protection of a Bacillus subtilis strain against grape downy mildew by both direct effect and defence stimulation
Source: Mol Plant Pathol. 2019 May 18;20(8):1037–50. doi: 10.1111/mpp.12809 (PMC6640177; doi:10.1111/mpp.12809)
Supplement: Supplementary file 1 — Table S1 Primers used for mutant construction. [file MPP-20-1037-s001.docx]

Table S1 Primers used for mutant construction

| **Primers** | **Sequence (5’-3’) (restriction sites underlined)** | **Amplicon** | **Reference** |
| --- | --- | --- | --- |
| *ppsB*-up- F (*Mlu* I) | CGACGCGTGTGAAATCAGATGCGCTCCA | *ppsB* deletion | This study |
| *ppsB*-up-R | AGCAGCCAGCCGGATG |  | This study |
| *ppsB*-dn-F | CGGCGGCACGATATTG |  | This study |
| *ppsB*-dn-R (*Bgl* II) | GAAGATCTGGTCCGCAGCAGGTAGTCTT |  | This study |
| *srfAA*-up- F (*Mlu* I) | CGACGCGTCTGTTCATCCGGTTATCCCT | *srfAA* deletion | This study |
| *srfAA*-up-R | TATCGTCTTGCCCGCTAAGTCCGACTCCCGCATCCT |  | This study |
| *srfAA*-dn-F | AGGATGCGGGAGTCGGACTTAGCGGGCAAGACGATA |  | This study |
| *srfAA*-dn-R (*Bgl* II) | GAAGATCTGCCAAGTTCTCCGCAGTTAC |  | This study |
