# Supplementary material for: Surfactin and fengycin contribute to the protection of a Bacillus subtilis strain against grape downy mildew by both direct effect and defence stimulation
Source: Mol Plant Pathol. 2019 May 18;20(8):1037–50. doi: 10.1111/mpp.12809 (PMC6640177; doi:10.1111/mpp.12809)
Supplement: Supplementary file 2 — Table S2 Primers used for qRT PCR. [file MPP-20-1037-s002.docx]

Table S2 Primers used for qRT-PCR

| **Primer** | **Targent gene** | **Accession No.** | **Sequence** | **Reference** |
| --- | --- | --- | --- | --- |
| EF1 ɣ F | elongation factor 1 chain gamma | AF176496 | GAAGGTTGACCTCTGGGATG | (Gamm *et al.*, 2011) |
| EF1 ɣ R |  |  | AGAGCCTCTCCCTCAAAAGG |  |
| PR1 F | pathogenisis related protein | [XM_002273752.1](http://www.ncbi.nlm.nih.gov/nucleotide/225429118?report=genbank&log$=nuclalign&blast_rank=2&RID=5D08TNKB01N) | CATGGGACAACACTGTGGCTG | (Hamiduzzaman *et al.*, 2005) |
| PR1 R |  |  | CCCAAGACGCACTGATTTGCT |  |
| PR2 F | class I beta-1,3-glucanase | [XM_002277133.1](http://www.ncbi.nlm.nih.gov/nucleotide/225431925?report=genbank&log$=nucltop&blast_rank=1&RID=5D0SZMH801N) | ATGCTGGGTGTCCCAAACTCG | (Aziz *et al.*, 2003, Dubreuil-Maurizi *et al.*, 2010) |
| PR2 R |  |  | CAGAACAAACTGCGCAAACCGT |  |
| PR3 F | class IV chitinase (CHI4C) | [XM_002275480.1](http://www.ncbi.nlm.nih.gov/nucleotide/225434077?report=genbank&log$=nucltop&blast_rank=1&RID=5DEX7VAE01N) | GCAACCGATGTTGACATATCA | (Aziz et al., 2003) |
| PR3 R |  |  | CGTCGCCCTAGCAAGTGAG |  |
| STS F | stilbene synthase | FJ851185 | AGGAAGCAGCATTGAAGGCTC | (Trouvelot *et al.*, 2008) |
| STS R |  |  | TGCACCAGGCATTTCTACACC |  |
| JAZ1 F | protein TIFY 10A-like 9 | XM_002277121.1 | GGCGAGGGGACCGGAGAAGT | (Kelloniemi *et al.*, 2015) |
| JAZ1 R |  |  | TCGGGCGTGCCGTTTCCTTC |  |
| Lox9 F | linoleate 9S-lipoxygenase 5-like | [XM_002280615.1](http://www.ncbi.nlm.nih.gov/nucleotide/225450912?report=genbank&log$=nucltop&blast_rank=2&RID=5FS1E3DB01N) | CTGGGTGGCTTCTGCTCTC | (Aziz et al., 2003, Dubreuil-Maurizi *et al*., 2010) |
| Lox9 R |  |  | GCATGAATCTGCGGCTTATC |  |
| PAL F | phenylalanine ammonia-lyase | XM_002268220 | AGTCTCCATGGACAACACCCG | (Aziz et al., 2003, Dubreuil-Maurizi *et al*., 2010) |
| PAL R |  |  | TGCTCAGCACTTTCGACATGG |  |

Reference:

Aziz, A., Poinssot, B., Daire, X., Adrian, M., Bezier, A., Lambert, B.*, et al.* (2003) Laminarin elicits defense responses in grapevine and induces protection against *Botrytis cinerea* and *Plasmopara viticola*. *Mol. Plant Microbe In.,* **16,** 1118-1128.

Dubreuil-Maurizi, C., Trouvelot, S., Frettinger, P., Pugin, A., Wendehenne, D. and Poinssot, B. (2010) beta-Aminobutyric acid primes an NADPH oxidase-dependent reactive oxygen species production during grapevine-triggered immunity. *Mol. Plant Microbe In.,* **23,** 1012-1021.

Gamm, M., Heloir, M. C., Kelloniemi, J., Poinssot, B., Wendehenne, D. and Adrian, M. (2011) Identification of reference genes suitable for qRT-PCR in grapevine and application for the study of the expression of genes involved in pterostilbene synthesis. *Mol. Genet. Genomics,* **285,** 273-285.

Hamiduzzaman, M. M., Jakab, G., Barnavon, L., Neuhaus, J. M. and Mauch-Mani, B. (2005) beta-Aminobutyric acid-induced resistance against downy mildew in grapevine acts through the potentiation of callose formation and jasmonic acid signaling. *Mol. Plant Microbe In.,* **18,** 819-829.

Kelloniemi, J., Trouvelot, S., Heloir, M. C., Simon, A., Dalmais, B., Frettinger, P.*, et al.* (2015) Analysis of the molecular dialogue between gray mold (*Botrytis cinerea*) and grapevine (*Vitis vinifera*) reveals a clear shift in defense mechanisms during berry ripening. *Mol. Plant Microbe In.,* **28,** 1167-1180.

Trouvelot, S., Varnier, A. L., Allegre, M., Mercier, L., Baillieul, F., Arnould, C.*, et al.* (2008) A beta-1,3 glucan sulfate induces resistance in grapevine against *Plasmopara viticola* through priming of defense responses, including HR-like cell death. *Mol. Plant Microbe In.,* **21,** 232-243.
